# LRRK2 G2019S mutation attenuates microglial motility by inhibiting focal adhesion kinase

**DOI:** 10.1038/ncomms9255

**Published:** 2015-09-14

**Authors:** Insup Choi, Beomsue Kim, Ji-Won Byun, Sung Hoon Baik, Yun Hyun Huh, Jong-Hyeon Kim, Inhee Mook-Jung, Woo Keun Song, Joo-Ho Shin, Hyemyung Seo, Young Ho Suh, Ilo Jou, Sang Myun Park, Ho Chul Kang, Eun-Hye Joe

**Affiliations:** 1 Department of Biomedical Sciences, Neuroscience Graduate Program, Ajou University School of Medicine, Suwon, Gyeonggi-do 443-380, Korea; 2 Department of Pharmacology, Ajou University School of Medicine, Suwon, Gyeonggi-do 443-380, Korea; 3 Chronic Inflammatory Disease Research Center, Ajou University School of Medicine, Suwon, Gyeonggi-do 443-380, Korea; 4 Department of Biochemistry and Biomedical Sciences, College of Medicine, Seoul National University, Seoul 110-799, Korea; 5 Bio Imaging and Cell Dynamics Research Center, School of Life Sciences, Gwangju Institute of Science and Technology, Gwangju 500-712, Korea; 6 Division of Pharmacology, Department of Molecular Cell Biology, Samsung Biomedical Research Institute, Sungkyunkwan University School of Medicine, Suwon, Gyeonggi-do 440-746, Korea; 7 Department of Molecular and Life Sciences, Hanyang University, Ansan 426-791, Korea; 8 Department of Physiology, Ajou University School of Medicine, Suwon, Gyeonggi-do 443-380, Korea; 9 Department of Brain Science, Ajou University School of Medicine, Suwon, Gyeonggi-do 443-380, Korea; 10 Brain Disease Research Center, Ajou University School of Medicine, Suwon, Gyeonggi-do 443-380, Korea

## Abstract

In response to brain injury, microglia rapidly extend processes that isolate lesion sites and protect the brain from further injury. Here we report that microglia carrying a pathogenic mutation in the Parkinson's disease (PD)-associated gene, G2019S*-LRRK2* (GS-Tg microglia), show retarded ADP-induced motility and delayed isolation of injury, compared with non-Tg microglia. Conversely, *LRRK2* knockdown microglia are highly motile compared with control cells. In our functional assays, LRRK2 binds to focal adhesion kinase (FAK) and phosphorylates its Thr–X–Arg/Lys (TXR/K) motif(s), eventually attenuating FAK activity marked by decreased pY397 phosphorylation (pY397). GS-LRRK2 decreases the levels of pY397 in the brain, microglia and HEK cells. In addition, treatment with an inhibitor of LRRK2 kinase restores pY397 levels, decreased pTXR levels and rescued motility of GS-Tg microglia. These results collectively suggest that G2019S mutation of *LRRK2* may contribute to the development of PD by inhibiting microglial response to brain injury.

## Abstract (web summary)

In response to brain injury, microglia extend processes to isolate the lesion. Here Choi *et al*. show that microglia expressing a pathogenic mutation in the Parkinson's disease-associated LRRK2 gene show reduced motility and delayed lesion isolation *in vitro* and *in vivo* due to attenuated focal adhesion kinase activity.

## Graphical abstract

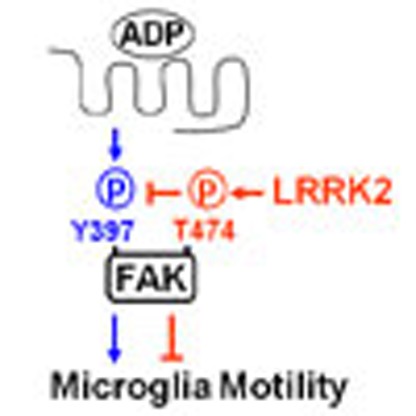

Leucine-rich repeat kinase 2 (*LRRK2*) is an autosomal dominant, late-onset familial Parkinson's disease (PD) gene. LRRK2 consists of several functional domains, including armadillo, ankyrin-like, leucine-rich repeat, Ras of complex proteins, C-terminal of Ras of complex proteins, kinase and WD40 domains, which may support diverse functions of LRRK2 (ref. 1). An important role of LRRK2 is regulation of actin dynamics; LRRK2 interacts with several actin-regulatory proteins, and regulates filopodia protrusion and neurite outgrowth^2^,^3^,^4^,^5^. PD-associated pathogenic mutations in LRRK2 are scattered throughout the entire *LRRK2* gene^6^. Of the mutations identified to date, G2019S has received the most attention since it is also found in sporadic PD^7^,^8^. G2019S is a pathogenic gain-of-function mutant that exhibits increased kinase activity, and thereby attenuates neurite outgrowth and increases neuronal death^9^,^10^,^11^.

Microglia, brain macrophages, use their highly branched processes to continuously scan the entire brain environment for abnormal structures and injury^12^,^13^,^14^. In response to brain injury, microglia rapidly extend processes to isolate lesion sites, preventing further injury that might be produced by disruptions in microenvironmental homeostatic^12^,^13^,^15^,^16^,^17^. Accordingly, it has been reported that, in the presence of an actin-depolymerizing agent, microglia do not properly isolate injury sites, worsening the damage^15^. Furthermore, defects in microglia have been reported in neurodegenerative diseases, including Alzheimer's disease and Huntington's disease^18^,^19^. In aged brains, microglial motility is also slowed^20^,^21^. Although microglia express LRRK2 (refs 22, 23), whether LRRK2 regulates microglial motility has not been studied.

Focal adhesion kinase (FAK) is a non-receptor tyrosine kinase that affects a range of cellular functions, including migration, proliferation, and survival^24^,^25^,^26^. FAK consists of an N-terminal FERM domain, a tyrosine kinase domain and a C-terminal focal adhesion targeting (FAT) domain^27^,^28^. It was originally reported that FAK is activated by the interaction between extracellular matrix and integrin^29^,^30^. FAK is also activated by several external stimuli, including activation of growth factor receptors or G protein-coupled receptors, and mechanical stress^31^,^32^,^33^,^34^. Although FAK is phosphorylated at multiple sites in response to stimuli, Y397 phosphorylation (pY397) is important for the proper integration of signalling pathways that control cell adhesion and migration^26^,^35^.

In this study, we report that LRRK2 is a negative regulator of microglial motility. LRRK2 inhibits FAK activation in a kinase-dependent manner, meaning that the G2019S gain-of-function mutation results in the excessive inhibition of FAK activation and microglial motility. Collectively, our findings suggest that the G2019S mutation of LRRK2 may prevent microglia from efficiently responding to brain damage, thereby contributing to the development of PD.

## Results

### The LRRK2 G2019S mutation retards microglial motility

**Figure 1:**
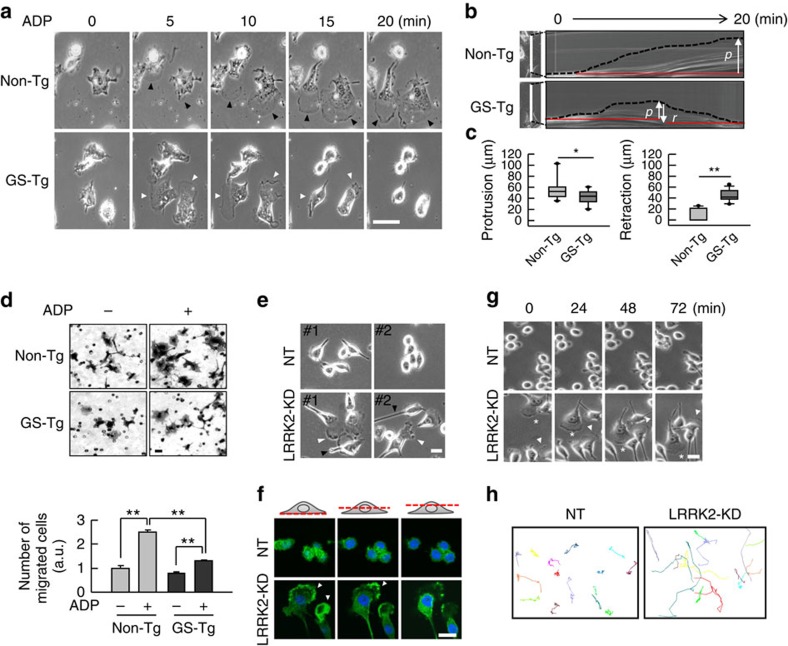
Microglial motility is retarded and accelerated, respectively, by the LRRK2 GS mutation and LRRK2 deficiency. (**a**) Time-lapse images of non-Tg and GS-Tg microglia were obtained every 5 s for 20 min after addition of 100 μM ADP. Black and white arrowheads represent lamellipodia. (**b**,**c**) Membrane dynamics of microglia (*n*=25) were quantified using SACED as described in the Methods section. Two-tailed Student's *t*-test, *P<0.05, **P<0.01. (**d**) Motilities of non-Tg and GS-Tg microglia were measured using transwells (upper panel). Number of cells migrated to the bottom of the transwells were counted at 12 h after plating (lower panel). Values are means±s.e.m. of three independent experiments. One-way ANOVA with Newman–Keuls *post hoc* test, **P<0.01. (**e**) Unlike NT microglia, LRRK2-KD microglia showed a morphological polarity with a leading edge corresponding to lamellipodia (white arrowheads) and a tail (black arrowheads) even in the absence of ADP. (**f**) NT and LRRK2-KD cells were stained with Alexa-488 phalloidin, and Z-stack scanned images were obtained at 2-μm intervals from the bottom. Lamellipodia attached to the bottom were enriched with F-actin in LRRK2-KD cells (arrowheads). (**g**) Time-lapse images of NT and LRRK2-KD cells were taken at 2-min intervals for 72 min. Asterisks and arrow heads chased two different cells that moved in the images. (**h**) Spontaneous migration paths of NT and LRRK2-KD cells (15 cells each) were tracked for 6 h. The location of each cell was determined every 10 min and connected to depict its migration route. Scale bar, 50 μm (**a**,**d**); 10 μm (**e**–**g**). Data are representative of at least three independent experiments unless indicated. ANOVA, analysis of variance.

In keeping with their role in continually surveying the brain microenvironment^12^,^14^, microglia have been reported to stretch their processes towards injured sites in response to purines, including ADP, ATP and UDP, released from damaged cells^13^,^36^. Since LRRK2 regulates actin dynamics^2^,^4^,^5^, we examined whether LRRK2 regulates microglial motility using microglia cultured from brain of *G2019S-LRRK2* transgenic (Tg) mice and littermate non-Tg mice. The morphologies of non-Tg and GS-Tg microglia were similarly diverse and indistinguishable (Fig. 1a). Non-Tg microglia rapidly (within 5 min) responded to ADP by forming lamellipodia (black arrowheads in Fig. 1a), a marker of migrating cells, and moving cell bodies for about 20 min (Fig. 1a; Supplementary Movie 1). Interestingly, however, GS-Tg microglia formed lamellipodia that rapidly shrunk by 10–15 min (white arrowheads in Fig. 1a). Furthermore, GS-Tg microglia barely moved in response to ADP (Fig. 1a; Supplementary Movie 1). Quantitative analyses using stroboscopic analysis of cell dynamics (SACED) showed that in response to ADP, GS-Tg microglia produced short protrusions (*p*) that were immediately retracted (*r*), whereas non-Tg cells exhibited long and wide protrusions (*p*) that rarely retracted (*r*) (Fig. 1b,c). In addition, we measured cell size reflecting the extent of lamellipodia protrusion from non-Tg and GS-Tg microglia in response to ADP. Consistent with SACED analysis, GS-Tg microglia exhibited significant decreased cell size from 5 to 20 min compared with non-Tg microglia (Supplementary Fig. 1), suggesting that GS-Tg microglia do not form lamellipodia in the presence of ADP. In migration assays using Transwells, GS-Tg microglia exhibited retarded migration compared with non-Tg microglia (Fig. 1d). On the basis of a previous report that ADP induces the movement of microglia through P2Y_12_ receptor^36^, we further used RT–PCR and immunostaining to examine P2Y_12_ receptor levels in non-Tg and GS-Tg microglia. However, we observed little difference in this parameter (Supplementary Fig. 2).

Next, we examined whether *LRRK2* knockdown (KD) affected the morphology and motility of microglia by comparing *LRRK2*-KD BV2 microglia and control cells prepared using *LRRK2*-targeted and non-targeted (NT) small hairpin RNAs (shRNAs), respectively^22^. Two different *LRRK2*-KD clones were tested and both showed a morphology typical of migrating cells flat and polarized (white and black arrowheads), and firmly attached to the substratum, whereas NT cells were weakly attached to the substratum and harboured short processes with relatively round shapes (Fig. 1e). *LRRK2*-KD cells stained with Alexa-488 phalloidin showed a dense F-actin structure at the leading edge compared with NT cells (Fig. 1f). Furthermore, *LRRK2*-KD cells moved much faster than NT cells, even in the absence of any activators (Fig. 1g). ‘Chase' experiments, in which individual NT and *LRRK2*-KD cells were followed for 6 h, revealed the obviously more rapid movement of *LRRK2*-KD cells (Fig. 1h; Supplementary Movie 2). Moving velocities of NT cells and *LRRK2*-KD cells were 7.5±0.4 × 10^−3^ μm s^−1^ and 14.5±1.5 × 10^−3^ μm s^−1^ (mean±s.e.m. of 15 cells), respectively. These data suggest that LRRK2 negatively regulate microglia motility *in vitro*.

**Figure 2:**
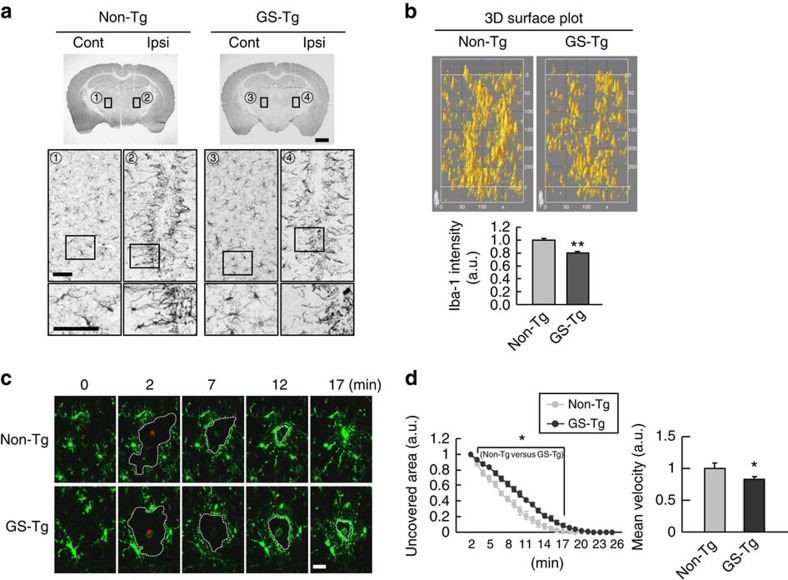
GS-Tg microglia slowly and inefficiently respond to brain injury. (**a**) Stab-wound injuries were produced in the striatum of non-Tg and GS-Tg mice, and microglia were analysed with Iba-1 antibodies at 1 h post injury. The most highly damaged sections were photographed. The middle and lower panels show higher magnifications of the areas boxed in the upper and middle panels, respectively. The contralateral sides (cont) were used as uninjured controls. (**b**) On the basis of the Iba-1 intensities obtained from the results shown in the middle panels of **a**, 3D surface plots were obtained and quantified using Image J software. Values are given as the means±s.e.m. of four animals. (**c**) GS-Tg microglia respond more slowly to laser-induced injury compared with non-Tg microglia. Laser injury was applied at 2 min to the cortices of 3-month-old non-Tg and GS-Tg mice expressing GFP in their microglia, and the behaviour of microglia was monitored by time-lapse imaging for at least 25 min. Asterisks: the sites laser injury was applied. (**d**) For measurement of the microglial response to injury, Image Analysis Software (Perkin-Elmer) was used to analyse the areas that were not covered by microglia (left panel) and the velocity of microglial processes moved towards the injury sites (right panel). Values are given as the means±s.e.m. of four animals. Two-tailed Student's *t*-test, *P<0.05; **P<0.01. Scale bars, 1 mm (**a**, upper panel); 100 μm (**a**, middle and lower panels); 20 μm (**c**).

Since microglia rapidly respond to injury and isolate damaged sites^13^,^15^, we used stab-wound and laser-induced injury models to examine the microglial response in non-Tg and GS-Tg mice. In the stab-wound injury model, Iba-1-positive microglia rapidly surrounded the injury sites within 1 h regardless of the tested genotype (Fig. 2a). However, microglia in GS-Tg mice isolated the injury sites less tightly compared with those in non-Tg mice (see 2 and 4 in Fig. 2a). The Image J software was used to quantify the Iba-1-positive pixels around the injury sites (Fig. 2b, upper panel), and the Iba-1 intensities were quantified (Fig. 2b, lower panel). Moreover, microglia in intact non-Tg and GS-Tg brains did not significantly differ in their morphologies or densities (see 1 and 3 in Fig. 2a). These results suggest that GS-Tg microglia are less active in their response to brain injury.

Next, we compared the responses of non-Tg and GS-Tg microglia to laser-induced damage using two-photon-live imaging. To generate non-Tg and GS-Tg mice that expressed green fluorescent protein (GFP) in their microglia, we crossed GS-Tg heterozygous mice (*GS/*-) with Cx3cr1–GFP (*GFP*/*GFP*) mice, as previously described^12^. Upon laser injury, both non-Tg and GS-Tg microglia extended their processes towards damaged areas, isolating and covering them within 20 min (Fig. 2c). However, GS-Tg microglia showed a delayed tendency for wound isolation (Fig. 2c,d). Thus, our results collectively indicate that GS-Tg microglia are retarded in their ability to respond to brain injury, delaying the isolation of injured sites with respect to surrounding tissues.

### LRRK2 interacts with FAK and inhibits its activation

**Figure 3:**
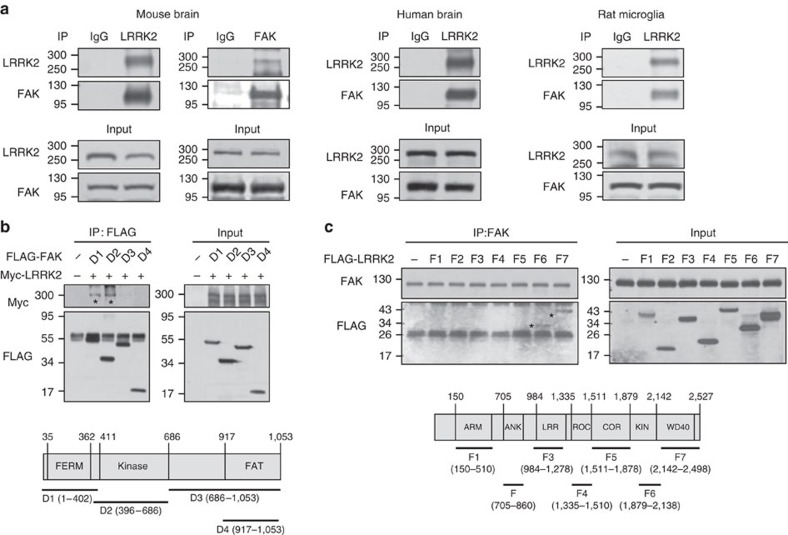
LRRK2 interacts with FAK. (**a**) Lysates were prepared from mouse and human brain, or rat primary microglia and immunoprecipitated (IP) using antibodies specific for LRRK2 and FAK. Immunoprecipitated proteins were analysed by western blot using antibodies specific for LRRK2 and FAK. IgG was used as a negative control. Levels of each protein in lysates were analysed using the indicated antibodies (input). (**b**,**c**) HEK 293T cells were transfected with Myc-tagged LRRK2 and FLAG-tagged FAK domains (D1–D4, lower panel) (**b**) or FLAG-tagged LRRK2 domains (F1–F7, lower panel) (**c**). Cell lysates were prepared 48 h after transfection, and immunoprecipitation was carried out using anti-FLAG (**b**) or anti-FAK (**c**) antibodies; proteins in IP complexes were identified by western blotting. Inputs in **b** and **c** show the amount of each protein used for IP. * indicates LRRK2 (**b**) or LRRK2 domains (**c**) that interacted with FAK. Data are representative of at least three independent experiments.

Next, we examined how LRRK2 reduced microglial motility. Since LRRK2 is known to regulate actin dynamics^5^, we examined actin-related proteins that interact with LRRK2. In immunoprecipitation assays using mouse brain lysates, we detected LRRK2–Rac1 interactions, as previously reported^2^, but found little interaction of LRRK2 with other actin-related proteins, including paxillin, VASP (vasodilator-stimulated phosphoprotein), PAK (p21 protein-activated kinase 1) and cofilin (Supplementary Fig. 3). Interestingly, LRRK2 co-immunoprecipitated with FAK, a key regulator of cell migration, in mouse and human brain lysates and rat microglial lysates (Fig. 3a). We then determined which domains of FAK and LRRK2 were required for their interaction. HEK 293T cells were transfected with full-length Myc-LRRK2 and FLAG-tagged domains of FAK, including FERM, kinase and FAT domain, designated D1–D4 (Fig. 3b). In immunoprecipitation assays using anti-FLAG antibodies, LRRK2 bound to FERM and kinase domains of FAK but not to the FAT domain (Fig. 3b). We also found that kinase and WD40 domains of LRRK2 expressed in HEK 293T cells interacted with FAK (Fig. 3c). Therefore, these results suggest that LRRK2 and FAK directly bind each other through kinase and WD40 domains of LRRK2 and FERM and kinase domains of FAK.

**Figure 4:**
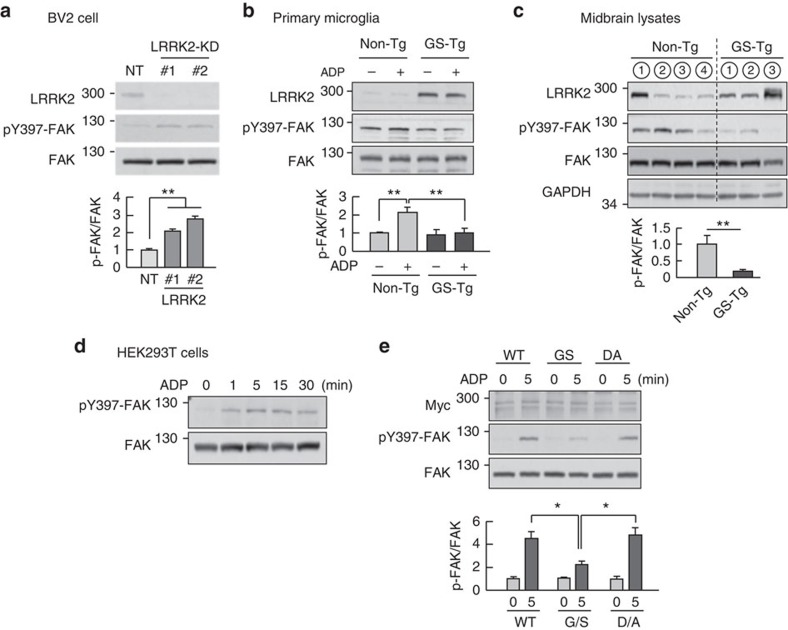
LRRK2 negatively regulates FAK activation. (**a**) The effect of LRRK2 on FAK activation was analysed by western blot using antibodies specific for pY397-FAK, FAK and LRRK2 (upper panels) in LRRK2-KD cells (#1 and #2) and a control (NT) cells used in Fig. 1. Band intensities of pY397-FAK were quantified, normalized to that of FAK and plotted (lower panels). Values are means±s.e.m. of three separate experiments. (**b**) Microglia cultured from non-Tg or GS-Tg mouse brains were treated with 100 μM ADP for 5 min, and the levels of pY397-FAK, FAK and LRRK2 were analysed. Values are means±s.e.m. of five separate experiments. (**c**) Midbrain lysates were prepared from 8-week-old GS-Tg mice and littermate non-Tg mice. Each number indicates a different animal. Values are means ± s.e.m. of three or four animals. (**d**) HEK 293T cells were treated with ADP (100 μM) for the indicated times, and the levels of pY397-FAK were measured. (**e**) HEK 293T cells were transfected with Myc-tagged WT-LRRK2, GS-LRRK2 and DA-LRRK2 mutants. Forty-eight hours later, cells were treated with ADP (100 μM) for the indicated times. Data are representative of three independent experiments. Values are means±s.e.m. of three independent experiments. One-way ANOVA with Newman–Keuls *post hoc* test, *P<0.05, **P<0.01 in **a**,**b** and **e**. Two-tailed Student's *t*-test, **P<0.01 in **c**. ANOVA, analysis of variance.

Next, we examined whether LRRK2 regulates microglial movement through FAK activation. We first examined whether FAK mediates ADP-induced microglial motility. In rat microglia, ADP treatment rapidly (within 1–5 min) induced FAK activation, which was monitored based on the autophosphorylation of FAK (pY397)^37^. Thereafter, the levels of pY397 decreased to the baseline at about 30 min (Supplementary Fig. 4a). Then, we treated two specific FAK inhibitors, PF573228 and FAK inhibitor 14 on microglia. These inhibitors have been known to be 50–250-fold more selective for FAK than for other kinases including Pyk2, CDK1/7, GSK-3β and receptor tyrosine kinases, EGFR, PDGFR and IGF-RI^38^,^39^. In the presence of inhibitors, these cells failed to form stable lamellipodia (Supplementary Fig. 4b) or showed decreased movement in the presence of ADP; in this, they were similar to GS-Tg microglia (Supplementary Fig. 4b–e versus Fig. 1a–d). Next, we found that pY397-FAK levels were higher in LRRK2-KD BV2 cells than in NT BV2 cells (Fig. 4a). Furthermore, ADP increased pY397-FAK levels to a lesser extent in GS-Tg microglia than in non-Tg microglia although basal pY397-FAK levels were little different (Fig. 4b). Interestingly, pY397-FAK levels were lower in midbrain lysates prepared from 8-week-old GS-Tg mice than in lysates prepared from age-matched, littermate control non-Tg mice (Fig. 4c). We further examined the effect of LRRK2 on Y397 phosphorylation by measuring pY397-FAK levels in HEK 293T cells in response to ADP, with or without transfection of various LRRK2 expression constructs. In untransfected HEK 293T cells, ADP induced an increase in pY397-FAK levels that reached a peaked at about 5 min (Fig. 4d). As was the case in microglial cells and brain lysates, the pathogenic GS-LRRK2 mutant significantly reduced pY397-FAK levels compared with WT-LRRK2 and the kinase dead mutant, D1994A (DA)-LRRK2, in response to ADP treatment (Fig. 4e). These results strongly suggest that LRRK2 regulates microglial motility through inhibition of FAK activation.

### LRRK2 phosphorylates TXR motifs in FAK.

**Figure 5:**
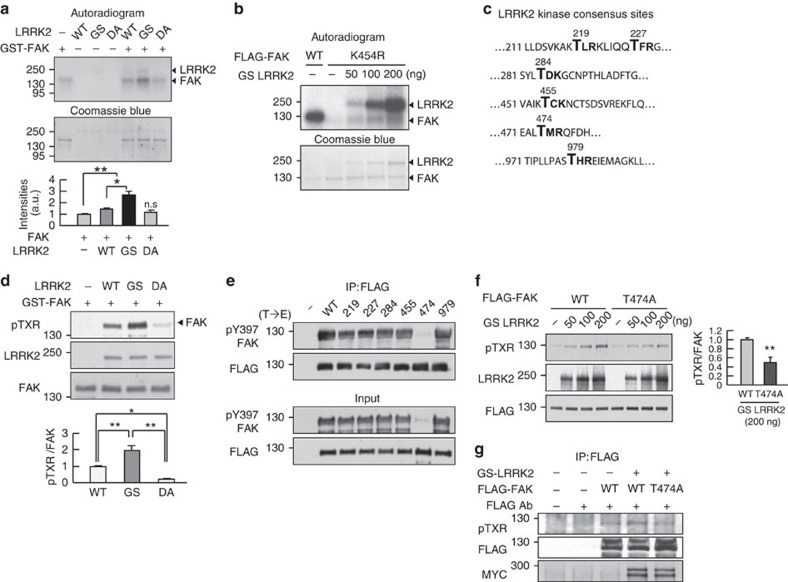
LRRK2 suppresses FAK Y397 phosphorylation through phosphorylation of TXR motif(s) in FAK. (**a**,**b**,**d**,**f**) *In vitro* kinase assays were carried out using 250 ng recombinant proteins of GST-FAK (**a**,**d**) FLAG-tagged WT, K454R mutant FAK (**b**) or T474A mutant FAK (**f**) and WT-, GS- and DA-LRRK2 recombinant proteins (50 ng in **a**,**d** or 50–200 ng in **b** and **f**) as indicated. ^32^P-labelled FAK was detected in autoradiograms (upper panels in **a** and **b**). Coomassie blue staining shows the amount of proteins in each reaction mixture (lower panels in **a** and **b**). In **d** and **f**, *in vitro* kinase assays were carried out using cold ATP without ^32^P-ATP, and the reaction mixtures were analysed by western blot using antibodies specific for FLAG, pTXR, FAK and LRRK2. Values are means±s.e.m. of three separate experiments. Data are representative of three independent experiments. (**c**) TXR(K) phosphorylation motifs found in FAK are indicated in bold. (**e**,**g**) Thr residues in six TXR sites (each number indicates the amino acid number in **c**) were mutated to Glu (T→E) (**e**). HEK 293T cells were transfected with FLAG-tagged WT-FAK or six (T→E) FAK mutants (**e**) or FLAG-tagged WT-FAK or T474A mutant FAK with GS-LRRK2 (**g**) for 48 h. Whole-cell lysates were prepared and immunoprecipitated with anti-FLAG antibody, and the levels of pY397-FAK and FLAG (**e**) or pTXR, Myc and FLAG (**g**) were analysed by western blot. One-way ANOVA with Newman–Keuls *post hoc* test in **a** and **d** *P<0.05, **P<0.01. Two-tailed Student's *t*-test in **f**. **P<0.01. ANOVA, analysis of variance.

The next question arising was how LRRK2, a Ser/Thr kinase^1^, regulates phosphorylation of Y397-FAK. *In vitro* kinase assays using WT and mutant versions of recombinant LRRK2 proteins, and GST-FAK proteins showed phosphorylation of FAK in the absence of LRRK2 (Fig. 5a). In the presence of WT-LRRK2 or GS-LRRK2, the phosphorylation levels of FAK further increased, with GS-LRRK2 producing a greater effect (Fig. 5a). In contrast, DA-LRRK2 did not increase FAK phosphorylation beyond basal phosphorylation level (Fig. 5a). We further examined whether LRRK2 increased the phosphorylation capacity of FAK. *In vitro* kinase assay using K454R-FAK (KR-FAK), a kinase-dead FAK mutant^40^, revealed that GS-LRRK2 induced a concentration-dependent increase in the phosphorylation level of KR-FAK, whereas KR-FAK alone was barely phosphorylated (Fig. 5b). These results demonstrate that LRRK2 directly phosphorylates FAK, indicating that FAK is a substrate of LRRK2 kinase.

It has been reported that LRRK2 favours phosphorylation of threonine residues in the TXR(K) motif, a consensus target sequence of LRRK2 (refs 41, 42). We identified six TXR(K) consensus sequences in FAK (Fig. 5c), and examined whether LRRK2 phosphorylates the TXR(K) motif(s) by performing *in vitro* kinase assays using non-radiolabelled cold ATP followed by western analyses using pTXR-specific antibody (Fig. 5d). As expected, LRRK2 increased pTXR-FAK levels, exhibiting a rank order of effectiveness of GS>WT>DA (Fig. 5d). We next used site-directed mutagenesis to introduce a phospho-mimetic glutamate (Glu, E) residue in place of the Thr residue in each TXR(K) motif of FLAG-tagged FAK to mimic LRRK2-induced phosphorylation. After expressing the mutant constructs in HEK 293T cells, we immunoprecipitated FAK with FLAG antibodies and measured the pY397-FAK levels of each mutant. Interestingly, replacement of T474 with Glu (T474E) strongly suppressed FAK Y397 phosphorylation (Fig. 5e). However, replacement of Thr residues in other TXR motifs had no effect on FAK Y397 phosphorylation (Fig. 5e). We further examined whether LRRK2 directly phosphorylated FAK T474 by comparing TXR phosphorylation levels of WT-FAK and T474A-FAK. To this end, we expressed FLAG-tagged WT-FAK and T474A-FAK in HEK 293T cells, immunoprecipitated them with FLAG antibody, and then carried out *in vitro* kinase assays using recombinant GS-LRRK2 protein (Fig. 5f). We also co-transfected HEK 293T cells with FLAG-tagged WT-FAK and T474A-FAK with GS-LRRK2. pTXR levels of FAK were analysed after immunoprecipitation using FALG antibody followed by western blot using pTXR antibody (Fig. 5g). GS-LRRK2 enhanced pTXR level of WT-FAK but not that of T474A-FAK in both conditions (Fig. 5f,g). Taken together, these results suggest that LRRK2 directly phosphorylate FAK at T474.

### LRRK2 interacts with FAK in the cytoplasm

**Figure 6:**
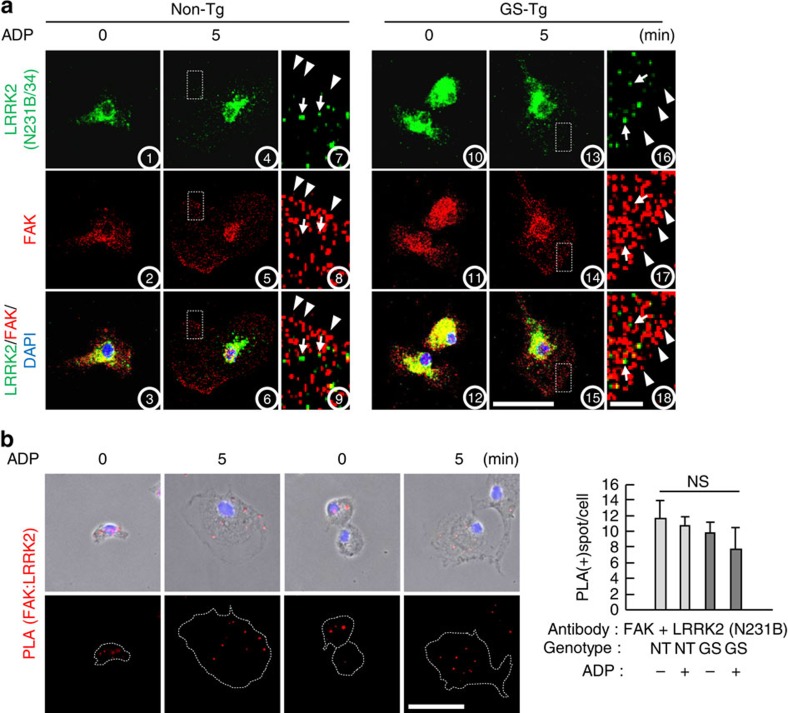
LRRK2 interacts with FAK in the cytoplasm. (**a**,**b**) Non-Tg and GS-Tg microglia were treated with 100 μM ADP for 5 min. (**a**) Cells were stained with specific antibodies against FAK and LRRK2 (N231B/34). Magnified images of boxed areas in 4–6 and 13–15 were shown in 7–9 and 16–18, respectively. Arrows and arrowheads indicate location of LRRK2 and FAK, respectively. (**b**) The interaction between FAK and LRRK2 was analysed in non-Tg and GS-Tg microglia (*n*=50) using *in situ* PLA, as described in the Methods section. White dotted lines indicate the cell boundaries (**b**, lower panel). PLA spots (representing interactions between the two proteins) were quantified (**b,** right panel). Values are means±s.e.m. of three separate experiments. NS, not significant. Scale bars, 50 μm (**a**, 15; **b**); 5 μm (**a**, 18).

**Figure 7:**
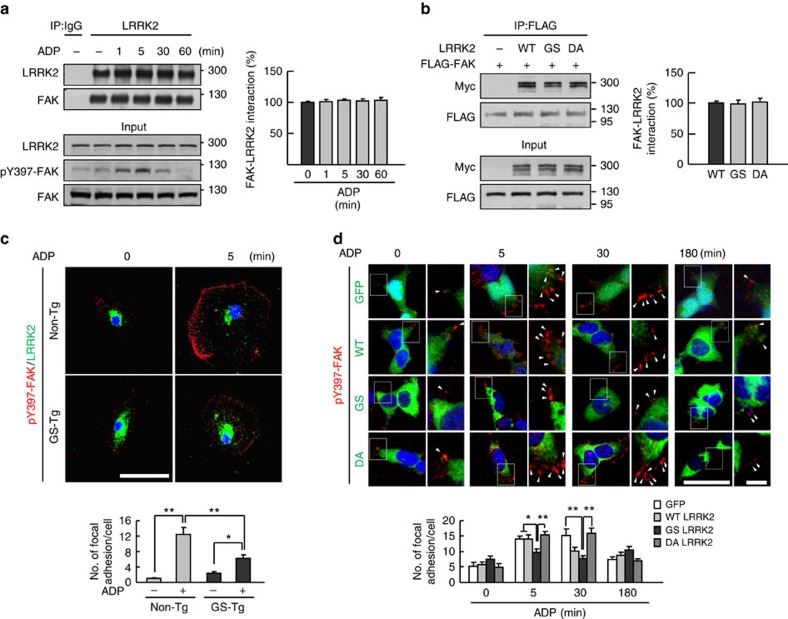
Effect of ADP and LRRK2 mutation on the LRRK2-FAK interaction and focal adhesion formation. (**a**) ADP does not affect the interaction between LRRK2 and FAK. HEK 293T cells were treated with 100 μM ADP for the indicated times, harvested and immunoprecipitated with an antibody against LRRK2. The amounts of FAK that co-immunoprecipitated with LRRK2 were analysed by western blot using antibodies specific for LRRK2 and FAK (left panel), and the results were quantified (right panel). IgG was used as a negative control. The levels of LRRK2 and pY397-FAK in lysates were analysed by western blot (input). (**b**) LRRK2 mutation does not affect the LRRK2-FAK interaction. HEK 293T cells were transfected with vectors encoding FLAG-FAK and Myc-LRRK2 (WT, GS or DA). Immunoprecipitation was performed using FLAG antibodies, the amounts of immunoprecipitated LRRK2 were analysed with western blot, and the results were quantified. (**c**,**d**) Non-Tg and GS-Tg microglia (*n*=25) (**c**) and HEK 293T cells transfected with Myc-LRRK2 (WT, GS or DA) vectors (*n*=50) (**d**) were treated with 100 μM ADP for the indicated times, fixed and stained with antibodies against pY397-FAK and LRRK2, and pY397-FAK and GFP or Myc (WT, GS and DA), respectively. The pY397-FAK-positive focal adhesions were counted using the Image J software (lower panel). *P<0.05 and **P<0.01 by one-way ANOVA with the Newman–Keuls *post hoc* test. Scale bar, 50 μm (**b**). Data are representative of at least three independent experiments. ANOVA, analysis of variance.

**Figure 8:**
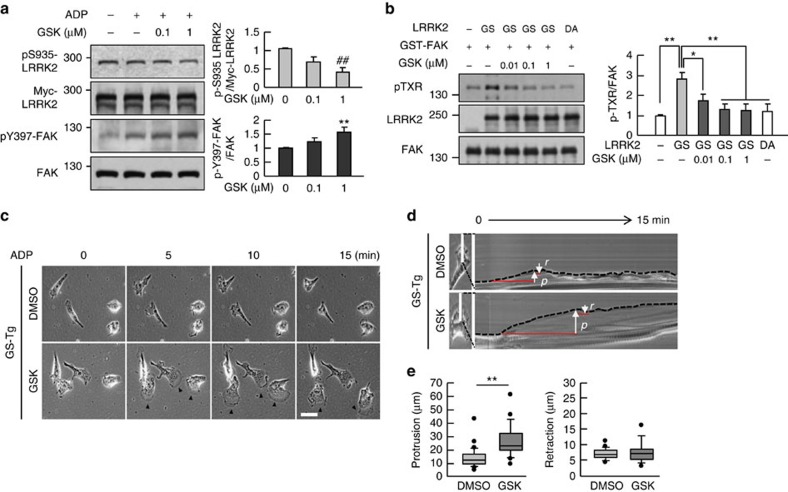
The LRRK2 kinase inhibitor, GSK2578215A (GSK), reduces pTXR-FAK levels and rescues pY397-FAK levels and motility of GS-Tg microglia. (**a**) HEK 293T cells expressing GS-LRRK2 were treated with ADP (100 μM) for 5 min in the presence of the indicated amount of GSK. Levels of S935-autophosphorylated LRRK2 (pS935-LRRK2) and Y397-autophosphorylated FAK (pY397-FAK) were measured by western blot using antibodies specific for pS935-LRRK2 and pY397-FAK, respectively. FAK and Myc were used as loading controls. Band intensities were quantified and plotted. Values are means±s.e.m. of three separate experiments. (**b**) *In vitro* kinase assays were carried out using recombinant GST-FAK and GS-LRRK2 (GS) or DA-LRRK2 (DA) in the absence or presence of the indicated amount of GSK. pTXR-FAK was analysed and plotted. Values are means±s.e.m. of three separate experiments. (**c**–**e**) GS-Tg microglia were treated with GSK (1 μM) for 30 min and then treated with ADP. ADP (100 μM) induced formation of stable lamellipodia followed by cell body movement in the presence of GSK (**c**, arrowheads). SACED showed that GSK (*n*=26) increased protrusion compared to DMSO (*n*=32) (**d**,**e**). One-way ANOVA with Newman–Keuls *post hoc* test, *P<0.05, **P<0.01, ^##^*P*<0.01 in **a** and **b**. Two-tailed Student's *t*-test, **P<0.01 in **e**. Scale bar, 50 μm (**c**). ANOVA, analysis of variance.

**Figure 9:**
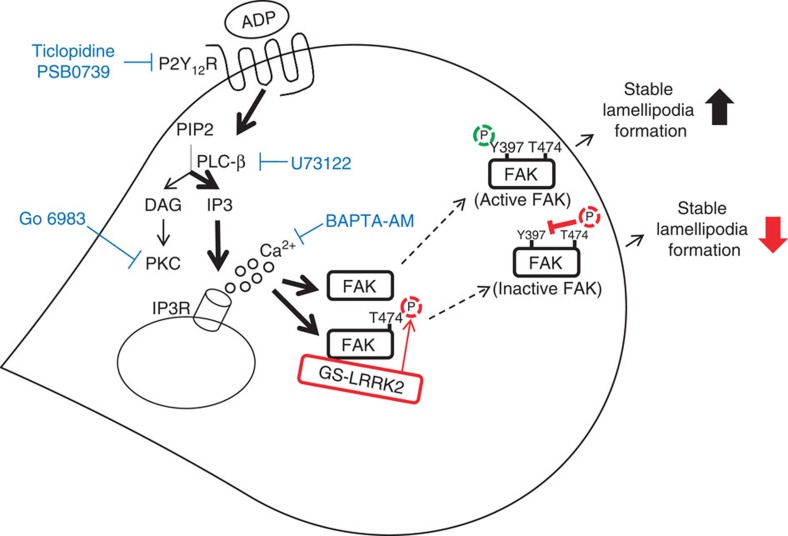
LRRK2 regulates microglial motility through inhibition of focal adhesion kinase. In response to ADP, microglia rapidly form lamellipodia and become highly motile. In this process, ADP activates FAK (demonstrated by Y397 phosphorylation) by activation of P2Y_12_ receptors and their downstream PLC-β, and intracellular Ca^2+^ release. Activated pY397-FAK is localized to leading edge of the cell and induces stable lamellipodia formation for proper cell migration. LRRK2 directly interacts and phosphorylates FAK on T474 in TXR motif, which prevents phosphorylation of FAK Y397. GS-LRRK2 excessively inhibits FAK Y397 phosphorylation with its enhanced kinase activity, resulting in unstable lamellipodia formation and improper migration.

Next, we examined where the two proteins interacted using two LRRK2-specific antibodies, N231B/34 (Fig. 6) and N241A/34 (Supplementary Fig. 5) that did not produce LRRK2 signal in LRRK2 knockout neurons^43^. In non-Tg and GS-Tg microglia cultured, LRRK2 was found in the cytoplasm near the nucleus in the absence of ADP (see 1 and 10 in Fig. 6a and 1 and 10 in Supplementary Fig. 5a), while FAK was located in both the cytoplasm and the nucleus (see 2, 3, 11 and 12 in Fig. 6a and 2, 3, 11 and 12 in Supplementary Fig. 5a). Following ADP treatment, FAK was obviously detectable in the lamellipodia (see 5, 6, 14 and 15 in Fig. 6a and 5, 6, 14 and 15 in Supplementary Fig. 5a) while most of LRRK2 remained in the cytoplasm near the nucleus (see 4, 6, 13 and 15 in Fig. 6a and 4, 6, 13 and 15 in Supplementary Fig. 5a). In magnified figures, a small portion of LRRK2 was found in the lamellipodia (see arrows present in 7–9 and 16–18 in Fig. 6a and 7–9 and 16–18 Supplementary Fig. 5a), but not at the edge of it where FAK was abundantly detectable (see arrowheads present in 7–9 and 16–18 in Fig. 6a and 7–9 and 16–18 Supplementary Fig. 5a). In addition, LRRK2 was not co-localized with FAK in the lamellipodia (see 9 and 18 in Fig. 6a and 9 and 18 in Supplementary Fig. 5a). Using a proximity ligation assay (PLA), we further examined interaction between LRRK2 and FAK. As shown in immunostaining (Fig. 6a), PLA spots were not detectable in the leading edge (Fig. 6b; Supplementary Fig. 5b), suggesting the interaction between two proteins mainly occurred in the cytoplasm. In addition, a portion of LRRK2 regardless of WT and GS appeared to consistently interact with FAK since the amounts of spots were not changed by ADP treatment and GS mutation (Fig. 6b; Supplementary Fig. 5b,c). Furthermore, locations of spots were not limited to the perinuclear region where LRRK2 was densely located, spots were also found in the region where LRRK2 was sparsely located (Fig. 6b; Supplementary Fig. 5b), indicating that the amount of LRRK2 minimally affected interaction with FAK. In control experiments with single primary antibodies or without any antibodies, PLA spots were barely detectable (Supplementary Fig. 5b,c).

Using HEK 293T cells, we further analysed whether the LRRK2 mutation altered the interaction between FAK and LRRK2. The interaction was detectable in the cytoplasm in the absence or presence of ADP (Supplementary Fig. 6a); the amount of FAK that co-immunoprecipitated with LRRK2 was not changed by the absence/presence of ADP regardless of FAK activation (as demonstrated by Y397 phosphorylation; Fig. 7a). Furthermore, similar amounts of LRRK2 WT, GS mutant and DA mutant co-immunoprecipitated with FAK (Fig. 7b). Next, we examined whether these LRRK2 mutations affected the formation of pY397-FAK-positive focal adhesions in non-Tg, GS-Tg microglia and HEK 293T cells (Fig. 7c,d). ADP treatment rapidly (within 5 min) increased pY397-FAK-positive focal adhesion in the lamellipodia in non-Tg microglia, but not in GS-Tg microglia (Fig. 7c). In HEK 293T cells, following ADP treatment, pY397-FAK-positive focal adhesions were formed at the cell edges within 5 min. These adhesions were maintained at 30 min but disappeared by 180 min in GFP-mock (GFP)-, WT-LRRK2 (WT)- and DA-LRRK2 (DA)-transfected cells (Fig. 7d). Similar with GS-Tg microglia, however, GS-LRRK2 (GS)-transfected cells showed significantly fewer pY397-FAK-positive focal adhesions (Fig. 7d). Taken together, these results indicate that LRRK2 regulates the cytoplasmic activation of FAK, and suggest that the enhanced inhibitory effect of the LRRK2 G2019S mutant on FAK activation may not be due to an increased interaction between the two proteins.

### Effect of LRRK2 inhibitor on FAK and microglial motility

Next, we examined whether inhibition of LRRK2 kinase activity rescued pY397-FAK levels using GSK2578215A (GSK), a LRRK2 inhibitor^44^. In GS-LRRK2-expressing HEK 293T cells, phosphorylation of LRRK2 at S935 (pS935) was decreased with GSK about 60%, confirming that this inhibitor effectively reduced LRRK2 kinase activity, as previously reported^44^ (Fig. 8a). We initially chose two well-known LRRK2 kinase inhibitors, IN-1 (ref. 45) and CZC 54254 (ref. 46). However, IN-1 and CZC 54254 have off-target effects on FAK^45^,^46^, whereas GSK has no such effect^44^. In our *in vitro* kinase assay also showed that IN-1 and CZC 54254 strongly suppressed basal phosphorylation of FAK in a dose-dependent manner (Supplementary Fig. 7a). Accordingly, these inhibitors did not rescue pY397-FAK in GS-LRRK2-expressing cells (Supplementary Fig. 7b). On the other hand, GSK induced increase in pY397-FAK levels while decreasing pS935 levels (Fig. 8a). *In vitro* kinase assays also showed that GSK eliminated the increase in pTXR-FAK levels induced by GS-LRRK2, reducing them to the levels of DA-LRRK2 or GST-FAK only (Fig. 8b). Moreover, GSK also changed microglial responses to ADP, markedly increasing the formation of stable lamellipodia in GS-Tg microglia (arrowheads in Fig. 8c and Supplementary Movie 3). SACED analysis further showed that GSK significantly increased protrusion but not affected retraction (Fig. 8e). Similar to GSK, GW5074, another inhibitor that potentially inhibits LRRK2 kinase activity^47^, inhibited the effects of GS-LRRK2: it increased pY397-FAK levels in GS-LRRK2-overexpressing HEK 293T cells, decreased pTXR levels produced by GS-LRRK2 *in vitro* kinase assay, rescued defect of stable lamellipodia formation, and increased cell migration (Supplementary Fig. 8; Supplementary Movie 4). Although GW5074 has been developed to inhibit Raf-1 kinase^48^, it is hard to think the effect of GW5074 on microglial motility and FAK is due to Raf-1 inhibition since Raf-1 inhibition rather retards cell motility^49^. Taken together, these results suggest that LRRK2 phosphorylates TXR motif(s) in FAK, which inhibits microglial motility through inhibition of Y397 phosphorylation. On the basis of this, we propose that the LRRK2 kinase inhibitors could potentially be used to treat and/or prevent PD by restoring the motility of G2019S microglia.

## Discussion

We herein report that microglia carrying the LRRK2 G2019S mutation (GS-Tg microglia) show retarded motility and fail to isolate injury sites as quickly and efficiently as non-Tg microglia. On the basis of results in this study, we proposed a model how LRRK2 and GS-LRRK2 regulate microglial motility (Fig. 9): when ADP binds to P2Y_12_ receptors, FAK is activated (marked by pY397-FAK) through PLCβ-, and Ca^2+^-dependent pathways (Supplementary Fig. 9), which is necessary for stable lamellipodia formation and proper microglial movement. In this process, LRRK2 negatively regulates microglial movement through direct interaction and phosphorylation of FAK on T474 in TXR motif, which prevents phosphorylation of FAK Y397. Mechanistically, G2019S-LRRK2 mutation having increased kinase activity retards microglial motility via the excessive inhibition of FAK activation (Fig. 9). Since the observed defect in the ability of these microglia to isolate injury sites will lead to worsening of damage^15^, our results collectively suggest that microglial defects caused by the G2019S mutation may contribute to the development of PD.

Since LRRK2 interacts with actin, and the actin-regulatory proteins^3^,^4^,^10^, we hypothesized that it could regulate microglial motility. Indeed, our results revealed that LRRK2 regulated microglial motility (Figs 1, 2). *LRRK2*-KD BV2 microglia were morphologically different from NT microglia, and highly motile even in the absence of any stimulators (Fig. 1e–h). Although GS-Tg microglia were not different from non-Tg microglia in morphology in culture and in intact brain (Figs 1a and 2a), these cells showed defects in response to ADP (Fig. 1a–c; Supplementary Fig. 1), or brain injury (Fig. 2). This suggests that the ability of LRRK2 to regulate microglial motility is related to its kinase activity. However, a recent study reported that GS-LRRK2 enhanced chemotactic responses of macrophages^50^. Fibroblasts derived from GS PD patients and R1441G mice also increased motility compared with those cells from non-PD patients and WT mice, respectively, through direct interaction with tubulin^51^. For microglial movement, actin dynamics is important^15^. Previously, it has been reported that actin and microtubule differently regulate movement of different types of cells^52^. Therefore, LRRK2 may differently regulate motility of cells depending on cell types and microenvironment of tissues.

We identified FAK as a substrate of LRRK2, demonstrating that FAK interacted with LRRK2 in cultured rat microglia, as well as mouse and human brain lysates (Fig. 3a). LRRK2 bound through its kinase and WD40 domains to FAK FERM and kinase domains (Fig. 3b,c). FAK has long been known as a critical player in processes that regulate cell movement^26^,^53^. In response to stimuli that induce motility of cells, FAK becomes autophosphorylated on Y397 (refs 54, 55, 56, 57), which is essential for lamellipodial formation and progression of migrating cells^58^. FAK is also phosphorylated on Y576/577, S732, Y861, Y863 and Y925 (refs 54, 55, 56, 57, 59). All of these phosphorylations enhance FAK activity and cell migration^54^,^55^,^56^,^57^,^59^. In this study, we provide a phosphorylation-induced negative regulatory mechanism of FAK. LRRK2-KD microglia showed higher levels of pY397 even in the absence of any stimulators (Fig. 4a). Conversely, microglia from Tg mice expressing the gain-of-function G2019S-LRRK2 mutant showed decreased pY397-FAK levels (Fig. 4b). This seemingly incongruous finding that the Ser/Thr kinase activity of LRRK2 is responsible for inhibiting FAK activation (Fig. 4) suggests that a complicated mechanism is involved in LRRK2-mediated inhibition of FAK Y397 phosphorylation. It has been reported that LRRK2 favours phosphorylation of Thr residue(s) in TXR(K) motifs^41^,^42^; six such sites are present in FAK (Fig. 5c). In a series of experiments, we found that LRRK2 phosphorylated at least one Thr residues in FAK TXR(K) motifs, namely Thr474, which in turn resulted in the inhibition of Y397 autophosphorylation. Replacing T474 in the FAK kinase domain with a phospho-mimic glutamate (T474E) completely eliminated FAK Y397 autophosphorylation (Fig. 5e); moreover, introduction of a non-phosphorylatable alanine at this same site (T474A) significantly decreased pTXR levels compared with that observed in WT-FAK in the presence of G2019S-LRRK2 (Fig. 5f,g), suggesting that T474 site is a LRRK2 target. Accordingly, a LRRK2 kinase inhibitor, GSK2578215A, increased pY397-FAK levels and reversed the motility phenotype of GS-Tg microglia (Fig. 8; Supplementary Movie 3). Therefore, LRRK2 appeared to decrease FAK Y397 phosphorylation through direct phosphorylation of at least one TXR/K motif (T474). However, we do not exclude possibilities that LRRK2 inhibits FAK Y397 phosphorylation through other mechanisms. Although kinase-dead DA-LRRK2 (ref. 60) did not increase pTXR to the same extent as WT-LRRK2 in *in vitro* kinase assays (Fig. 5d), pY397-FAK levels were not different in DA- and WT-LRRK2-expressing cells (Fig. 4e). Therefore, LRRK2 may decrease FAK Y397 phosphorylation through direct and/or indirect interaction with other signalling molecules that inhibit FAK Y397 phosphorylation such as phosphatases and/or suppressor of cytokine signalling proteins^61^,^62^,^63^,^64^.

The interaction between LRRK2 and FAK occurred mainly in the cytoplasm of microglia and HEK 293T cells (Fig. 6a,b; Supplementary Figs 5,6). Interestingly, this interaction showed little change in response to ADP treatment (Figs 6b and 7a; Supplementary Figs 5b and 6a) or the LRRK2 mutation (Figs 6b and 7b). In HEK 293T cells, however, the number of focal adhesions increased following ADP treatment (Fig. 7c,d); in the latter system, the GS and DA mutations, respectively, decreased and increased the number of focal adhesions compared with WT LRRK2 (Fig. 7d). Furthermore, when HEK cells were plated on fibronectin, LRRK2 was not located at focal adhesion strongly stained with FAK and paxillin (Supplementary Fig. 6b). Accordingly, FAK but not LRRK2 was located at the edge of lamellipodia (Fig. 6a; Supplementary Fig. 5a). On the basis of these results, we speculate that FAK free from LRRK2-mediated phosphorylation was phosphorylated on Y397 and positively regulates microglia motility and/or focal adhesion of HEK 293T cells (Fig. 9).

Next arising question was what is a physiological role for LRRK2 in modulating microglial motility. We speculate that LRRK2 inhibits unnecessary movement of cells, since LRRK2-KD BV2 cells were highly motile even in the absence of a stimulus (Fig. 1). Although continuous surveillance in the brain is an important microglial function^12^,^13^,^14^, excessive movement would entail the expenditure of unnecessary energy and effort. Therefore, LRRK2 may balance the surveillance function of microglia with the competing need to conserve energy. However, LRRK2 mutations such as the gain-of-function G2019S excessively inhibited microglial surveillance function (Figs 1 and 2), which may result in an inadequate response of microglia to brain injury and the accumulation of defects in the brain.

In conclusion, LRRK2 G2019S mutation leads to defects in microglia motility and response to injury. Studies on PD and other neurodegenerative diseases have focused on neurons. However, neuronal death can be induced not only by defects in neurons themselves but also by defects in glia including microglia, which support neuronal survival and function in diverse ways. Since PD-related genes such as *LRRK2*, *DJ-1*, *PINK1* and Parkin are expressed in glia, mutations or knockout of these genes alter functions of glia^22^,^65^,^66^,^67^,^68^. Our present findings that the pathogenic mutant, LRRK2 G2019S, causes defects in microglial motility are consistent with the microglial defects clearly observed in other neurodegenerative diseases, including Alzheimer's disease and Huntington's disease^18^,^19^.

## Methods

### Animals

G2019S-*LRRK2*-Tg FVB mice^69^ were purchased from Jackson Laboratory (stock #009609, Bar Harbor, ME, USA). Cx3cr1–GFP (*GFP/GFP*) mice were kindly gifted from Dr Seog Bae Oh (Seoul National University School of Dentistry, Seoul, Korea). Non-Tg and G2019S-*LRRK2*-Tg heterozygote mice were prepared by crossing G2019S-*LRRK2* heterozygote mice with wild-type FVB mice or Cx3cr1–GFP (*GFP/GFP*) mice^19^. Genotyping was carried out as described in manufacturer's instruction (Jackson Laboratory). All animal procedures were approved by the Ajou University Institutional Animal Experimentation Committee (AMC-119).

### Cell culture

Primary microglia were obtained from mixed glia cultured from cerebral cortices of 1-day-old Sprague Dawley rats (Samtako, Seoul, Korea), or wild type and G2019S-LRRK2 heterozygote mice as described previously^70^. Genotyping was carried out as described in manufacturer's instruction (Jackson Laboratory). Briefly, cortices were isolated and single-cell suspensions were prepared by triturating with Pasteur pipettes in Minimal Essential Medium (Sigma, St Louis, MO, USA) containing penicillin/streptomycin (100 U ml^−1^), 10 mM HEPES, 10% fetal bovine serum (FBS; HyClone), and 2 mM L-glutamine. Cells were plated at 75 cm^2^ T-flask (BD Bioscience, San Jose, CA, USA) and incubated for 2–3 weeks. Microglia were detached from flasks by shaking and then filtered through nylon mesh to remove other cells and cell clumps. Microglia were counted and plated onto culture dishes at an appropriate density. The BV2 murine microglia cell lines were grown in DMEM containing 4 mM L-glutamine, 20 mM HEPES (pH 7.4), penicillin/streptomycin (50 U ml^−1^) and 10% (v/v) FBS. BV2 clones LRRK2-KD and NT, stably expressing shRNA targeting LRRK2 (sc-45750-V; Santa Cruz Biotechnology, Santa Cruz, CA, USA) or non-targeting shRNA controls (sc-108080; Santa Cruz Biotechnology), respectively, were prepared by transfection with lentiviral particles and subsequent selection^22^. In detail, cells (5 × 10^4^ cells per well) were seeded in 12-well plates and incubated with polybrene (5 μg ml^−1^) and lentiviral particles (1.0 × 10^5^ infectious units) for 12 h. Infected cells were selected with puromycin (5 μg ml^−1^) for 2 days, and plated into 96-well plates at a density of 0.5 cells per well for clonal selection with puromycin. *LRRK2*-KD clones that expressed <20% of basal levels of Lrrk2 messenger RNA were chosen. The HEK 293T cell line was maintained in DMEM supplemented with 10% FBS.

### DNA constructs

Plasmid DNA for C-terminal 3xMyc-tagged wild-type LRRK2 (WT-LRRK2), G2019S-LRKK2 and D1994A-LRKK2 were kind gifts from Dr Wongi Seol (Wonkwang University, Gyeonggi-do, South Korea). FLAG-FAK was prepared by inserting coding sequences of the human *FAK* gene (NM_153831.3) into the p3xFLAG-CMV-7.1 vector (Sigma) using AccuPrime Pfx DNA Polymerase (Invitrogen, Carlsbad, CA, USA) and an infusion cloning kit (Clontech, Palo Alto, CA, USA). Inserts for FLAG-FAK fragments (D1–D4) and FLAG-LRRK2 fragment were prepared from FLAG-FAK and Myc-tagged WT LRRK2 vectors, respectively. Mutations were introduced into FLAG-FAK using a QuikChange Lightning Site-Directed Mutagenesis Kit (Agilent Technologies, Palo Alto, CA, USA). Primers used for mutations are listed in Supplementary Table 1.

### Transfection

HEK 293T cells were transfected with DNA plasmids using the jetPEI transfection reagent (Polyplus-Transfection, San Diego, CA, USA) as described by the manufacturer. Briefly, cells were exposed to DNA plasmids and jetPEI mixture for 4 h. Media were then replaced with fresh DMEM containing 10% FBS. Two days later, transfected cells were used for experiments.

### Time-lapse microscopy and SACED

Time-lapse images were obtained every 5 s for 15–20 min on an inverted microscope (Zeiss Xiovert 200) equipped with a × 20 objective. Stroboscopic images were generated in a 5 × 200-pixel-wide (5 s × 147 m) box drawn in the direction of cell protrusions using Metamorph software (Nashville, TN, USA). Box and whisker plots for the protrusion distance (*p*) and retraction distance (*r*) of individual events (*n*>20) were produced using SigmaPlot software. The middle line in each box indicates the median; the top of each box indicates the 75th percentile; the bottom indicates the 25th percentile; and the whiskers indicate the extent of the 5th and 95th percentiles.

### Migration assay

Microglia (10^5^ cells per well) were seeded onto 8-μm-pore Transwells (Corning, Inc., Lowell, MA, USA), and the Transwells were placed on a chamber containing 100 μM ADP. Cells were allowed to migrate for 4–12 h. Non-migrated cells were removed from the top surface of Transwells with a cotton swab. Cells that had migrated to the bottom surface of the Transwells were fixed with 4% paraformaldehyde for 20 min and visualized by staining with 1% Crystal violet (Sigma). The number of migrated cells in nine randomly chosen fields was counted. The FAK inhibitors, FAK 14 and PF573228 (Santa Cruz), were treated for 30 min before adding cells on top surface.

### *In vivo* stab-wound injury model and preparation of tissues

Male non-Tg and G2019S-LRRK2-Tg heterozygote mice (10 weeks old) were anaesthetized by Avertin (Sigma) and positioned in a stereotaxic apparatus (Kopf Instruments, Tujunga, CA, USA). Stab wound was produced with a 26-G needle in the right striatum (AP, −1.3 mm; ML, −1.2 mm; DV, −3.8 mm from the bregma) according to the atlas of Paxinos and Franklin. At 1 h after the injury, mice were anaesthetized and transcardially perfused with saline solution containing 0.5% sodium nitrate and heparin (10 U ml^−1^), and then with 4% paraformaldehyde in 0.1 M phosphate buffer (pH 7.4). Brains were obtained and post-fixed overnight at 4 °C in 4% paraformaldehyde. Fixed brains were stored at 4 °C in a 30% sucrose solution until they sank. Series of coronal sections (30 μm) were obtained with a cryostat (Leica, Wetzlar, Germany), and used for immunohistochemistry.

### Two-photon microscopic analysis

Mice expressing GFP in their microglia were obtained by crossing G2019S-LRRK2 heterozygous mice (*GS/-)* with Cx3cr1–GFP (*GFP/GFP*) mice. For open-skull craniotomy surgery, mice were anaesthetized with Zoletil 50 (Virbac, intramuscular injection, 30 μl), fixed on the stereotactic heating plate (Live Cell Instruments, Seoul, Korea). After removing the scalp and the periosteum, the region (1 mm from bregma, 1 mm from sagittal suture; diameter, 3 mm) of the skull was drilled with a microdrill. Then, the bone was removed elaborately. Three-millimetre-round coverslip was attached to the region with Loctite 454 (Loctite, Rock Hill, CT, USA). Last, emerged portions of the skull were covered with dental acryl. Multiphoton imaging was performed using a LSM 7 MP two-photon laser-scanning microscope (Carl Zeiss Microscopy GmbH, Oberkochen, Germany)^71^. Briefly, laser (920-nm wavelength, 30-mW intensity) was transiently applied to the brain (135-μm depth from the surface). The areas uncovered by microglia were measured, and plotted time-dependent changes in the area. Image Analysis Software (Perkin-Elmer, Waltham, MA, USA) was used to process the images and track the movements of microglia.

### Immunostaining

For 3,3′-diaminobenzidine staining, serial sections were rinsed three times with PBS, treated with 3% H_2_O_2_ for 5 min and rinsed with PBS containing 0.2% Triton X-100 (PBST). Non-specific binding was blocked with 1% bovine serum albumin in PBST. Sections were incubated overnight at room temperature with primary antibodies specific for Iba-1 (1:1,000, 019-19741, Wako). Following rinsing in PBST, sections were incubated with biotinylated secondary antibodies (Vector Laboratories, Burlingame, CA), and visualized according to the manufacturer's guidance. Sections were mounted on gelatin-coated slides, and examined under a bright-field microscope (Olympus Optical, BX51, Tokyo, Japan). Images were analysed using Image J (NIH, Bethesda, MD, USA).

For immunofluorescence staining, cells were seeded onto coverslips (Fisher Scientific Co., Fair Lawn, NJ, USA), fixed with 4% paraformaldehyde in a cytoskeleton stabilization buffer (10 mM MES (pH 6.1), 138 mM KCl, 3 mM MgCl_2_, 2 mM EGTA and 0.32 M sucrose), permeabilized with 0.1% Triton X-100 and treated with 1% bovine serum albumen. Cells were incubated with primary antibodies against LRRK2 (1:50, N231B/34 and N241A/34, NeuroMab), FAK (1:50, #3285, Cell Signaling), pY397-FAK (1:20, #3283, Cell Signaling), Myc-tag (1:500, #2278, Cell Signaling) and GFP (1:500, ab13970, Abam), and visualized with Alexa-488- and/or Alexa-555-conjugated secondary antibodies (1:500, A21202 and A31572, Invitrogen). Cells were incubated with Phalloidin-conjugated Alexa-488 (30 nM; Cytoskeleton Inc., Denver, CO, USA) used for F-actin staining. Cells were mounted with Vecta shield (Vector Laboratories). Images were captured with a confocal microscope (LSM510 Carl Zeiss, Oberkochen, Germany).

### Proximity ligation assay

Cells were fixed with 4% paraformaldehyde, permeabilized with 0.1% Triton X-100 and treated with 1% bovine serum albumen. Cells were then incubated with LRRK2 (1:50, N231B/34 and N241A/34, NeuroMab) and FAK (1:50, #3285, Cell Signaling) antibodies together and incubated with DNA probe-conjugated secondary antibodies (Olink Bioscience, Uppsala, Sweden) for 1 h at 37 °C. Cells were washed, and DNA probes were ligated for 30 min at 37 °C, amplified for 2 h at 37 °C, and examined under an Axiovert 200 M microscope (Carl Zeiss). Images were analysed using Image J (NIH, Bethesda, MD, USA).

### Western blot analysis

Cells and mouse brains were lysed on ice in RIPA buffer (50 mM Tris-HCl (pH 7.4), 1% NP-40, 1 mM NaF, 0.25% Na-deoxycholate, 1 mM Na_3_VO_4_ and 150 mM NaCl) containing protease/phosphatase inhibitor cocktail (GenDEPOT, Barker, TX, USA). Lysates were centrifuged, and proteins in the supernatant were separated by SDS–polyacrylamide gel electrophoresis and blotted onto nitrocellulose membranes (Protran, Schleicher & Schuell, Dassel, Germany). Membranes were incubated with antibodies specific for LRRK2 (1:1,000, ab133474, Abcam), pS935-LRRK2 (1:1,000, ab133450, Abcam), FAK (1:1,000, #3285, Cell Signaling), pY397-FAK (1:1,000, #3283, Cell Signaling), Myc (1:1,000, #2278, Cell Signaling), FLAG (1:1,000, F1804, Sigma), pTXR (1:1,000, #2351, Cell Signaling) and glyceraldehyde-3-phosphate dehydrogenase (GAPDH, 1:1,000, sc-48167, Santa Cruz). Membranes were washed with Tris-buffered saline containing 0.1% Tween 20, incubated with secondary antibodies and visualized with an enhanced chemiluminescence system (Daeil Lab Inc., Seoul, Korea). Uncropped scan for the main figures is presented in Supplementary Fig. 10.

### Immunoprecipitation assay

Cells and brain tissue were lysed with an immunoprecipitation buffer (1% Triton X-100, 150 mM NaCl, 10 mM NaH_2_PO_4_, 15 mM Na_2_HPO_4_, 50 mM NaF, 1 mM EDTA and 1 mM Na_3_VO_4_). Human brain lysates were purchased from Novus Biological (Littleton, CO, USA). Cell lysates and mouse and human brain lysates (200–500 μg) were incubated with primary antibodies against LRRK2 (1 μg, ab181386, Abcam), FAK (1 μg, sc-558, Santa Cruz), FLAG (1 μg, F1804, Sigma) and IgG (1 μg, #3900, Cell Signaling), and then with Protein G agarose beads (20 μl per a reaction, Millipore, Billerica, MA, USA). Beads were washed with immunoprecipitation buffer and boiled in 2 × sample buffer. Immunoprecipitated proteins were identified by western blot.

### *In vitro* kinase assay

For *in vitro* kinase assays, 50 ng of recombinant human GST-WT-LRRK2, GST-G2019S-LRRK2 or GST-D1994A-LRRK2 (Invitrogen) were incubated with 250 ng of recombinant human GST-FAK (Invitrogen) in kinase buffer S (50 mM Tris-HCl (pH 8.5), 10 mM MgCl_2_, 0.01% Brij-35 and 1 mM EGTA; Invitrogen) including protease/phosphatase inhibitor cocktail (GenDEPOT), 10 μM ATP and/or 1 μCi ml^−1 32^P-ATP (Perkin-Elmer-Cetus, Norwalk, CT, USA). For some experiments, WT and mutant FLAG-FAK constructs (FAK (T→E), K454R or T474A) were expressed in HEK 293T cells, immunoprecipitated, isolated from agarose beads and used for *in vitro* kinase assays. To release FLAG-FAK proteins from agarose beads, the protein–antibody–bead complex was treated with 100 μM FLAG peptide (Sigma) in 50 mM Tris-HCl (pH 7.4).

### Image analysis

Band intensities in western blots and Coomassie blue-stained gels were quantified using one-dimensional scan software (Scanalytics, Fairfax, VA, USA).

### Statistical analysis

The statistical significance of differences between two groups was determined using unpaired two-tailed Student's *t*-tests. For multiple-means comparisons, statistical significance was determined by one-way analysis of variance followed by Newman–Keuls *post hoc* test or two-way analysis of variance with Bonferroni *post hoc* test using Graph Pad Prism 5 (GraphPad Software, CA, USA).

## Additional information

**How to cite this article:** Choi, I. *et al*. LRRK2 G2019S mutation attenuates microglial motility by inhibiting focal adhesion kinase. *Nat. Commun.* 6:8255 doi: 10.1038/ncomms9255 (2015).

## Supplementary Material

Supplementary Information Supplementary Figures 1-10 and Supplementary Table 1

Supplementary Movie 1 non-Tg and G2019S-Tg microglia responded differently to ADP (100 μM). Images were acquired for 20 minutes using a Zeiss microscope equipped with a 20X objective. G2019S-Tg microglia exhibited sudden shrinkage of leading edges, indicating failure to form stable lamellipodia.

Supplementary Movie 2 NT and LRRK2-KD BV2 microglia exhibited different morphologies and motility. NT BV2 and LRRK2-KD BV2 microglia were followed for 6 hours using a microscope equipped with a 20X objective. LRRK2-KD BV2 microglia moved continuously compared with NT BV2 microglia, which were relatively stationary.

Supplementary Movie 3 The LRRK2 kinase inhibitor, GSK2578215A, altered the response of G2019S-Tg microglia to ADP. G2019S-Tg microglia were pre-incubated with DMSO or GSK2578215A (1 μM) for 30 minutes and then treated with ADP. Images were collected over the course of 15 minutes.

Supplementary Movie 4 The LRRK2 kinase inhibitor, GW5074, altered the response of G2019S-Tg microglia to ADP. G2019S-Tg microglia were pre-incubated with DMSO or GW5074 (10 μM) for 30 minutes and then treated with ADP. Images were collected over the course of 15 minutes.
