# Supplementary material for: LRRK2 G2019S mutation attenuates microglial motility by inhibiting focal adhesion kinase
Source: Nat Commun. 2015 Sep 14;6:8255. doi: 10.1038/ncomms9255 (PMC4647842; doi:10.1038/ncomms9255)
Supplement: Supplementary Information — Supplementary Figures 1-10 and Supplementary Table 1 [file ncomms9255-s1.pdf]

Supplementary Information

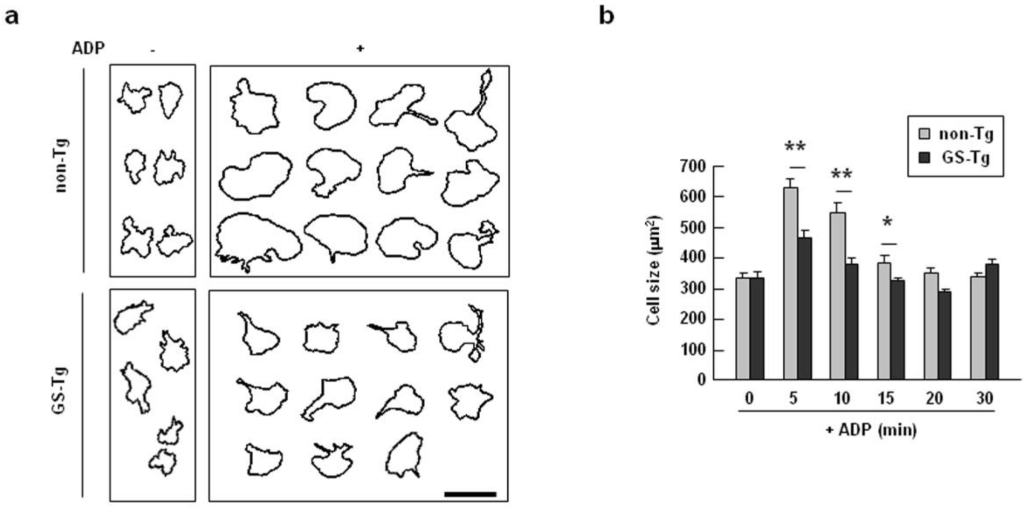

Supplementary Figure 1. Analysis of cell sizes of non-Tg and GS-Ts microglia in response to ADP. (a) Shapes of non-Tg and GS-Tg microglia were generated by tracing the outlines of cells with Photoshop. Scale bar, 50 μm. **(b)** Cell sizes of non-Tg and GS-Tg microglia (n > 50) were analyzed with Image J. Two-way ANOVA with Bonferroni *post-hoc* test, *, P<0.05; **P<0.01.

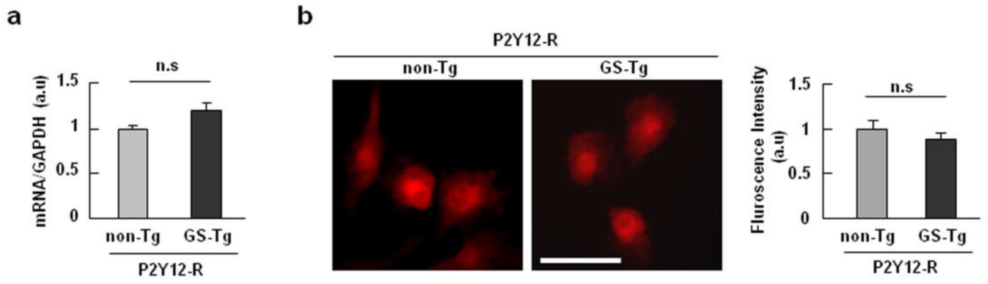

Supplementary Figure 2. Analysis of P2Y$_{12}$ levels in non-Tg and GS-Tg microglia. (a) Total RNA was isolated using RNAzol B (iNtRON, Sungnam, Korea), and cDNA was prepared using Avian Myeloblastosis Virus reverse transcriptase (Promega, Madison, WI, USA) according to the manufacturers' instructions. *P2Y$_{12}$* mRNA levels were measured using a KAP SYBR FAST qPCR kit (Kapa Biosystems, Boston, MA, USA) and a RotoGene thermocycler (Corbett Research, Sydney, Australia). The primer pairs (Intergrated DNA Technologies, Coralville, IA, USA) were 5'-GGTTCAGCCAAAGTTCCCAAGA-3' (forward) and 5'-CCGAGTTTGGCTCAGGGTGTA-3' (reverse) for *P2Y$_{12}$*, and 5'-GCCTTCCGTGTTCCTACC-3' (forward) and 5'-CCTCAGTGTAGCCCAAGATG-3'(reverse) for *GAPDH*. The cycle threshold (Ct) for the gene transcript was normalized to the average Ct for transcripts of the housekeeping gene, *GAPDH*, amplified in each reaction. Relative quantitation of normalized transcript abundance was determined using the comparative Ct method (ΔΔCt). **(b)** Non-Tg and GS-Tg microglia were fixed, stained with P2Y$_{12}$ specific antibodies (1:200, Alomone Laboratories, Jerusalem, Israel), and visualized with Alexa-555-conjugated secondary antibodies. Images were captured by a fluorescence microscope (Zeiss), and intensities were analyzed using Image J. Scale bar, 50 μm.

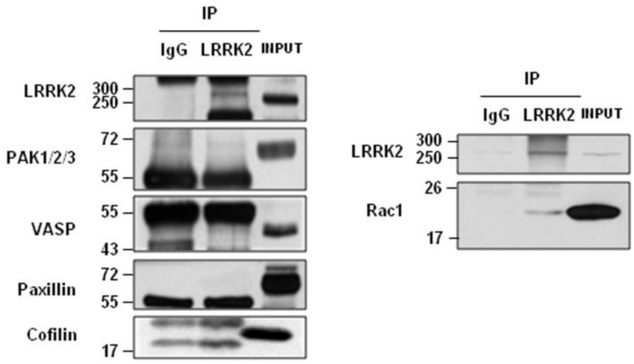

Supplementary Figure 3. Analysis of actin-interacting proteins that bound LRRK2. Mouse (FVB) brain lysates were immunoprecipitated with an LRRK2-specific antibody. Proteins that co-immunoprecipitated with LRRK2 were analyzed by Western blotting using antibodies specific for PAK1/2/3, VASP, paxillin, cofilin, and Rac1. IgG was used as a negative control.

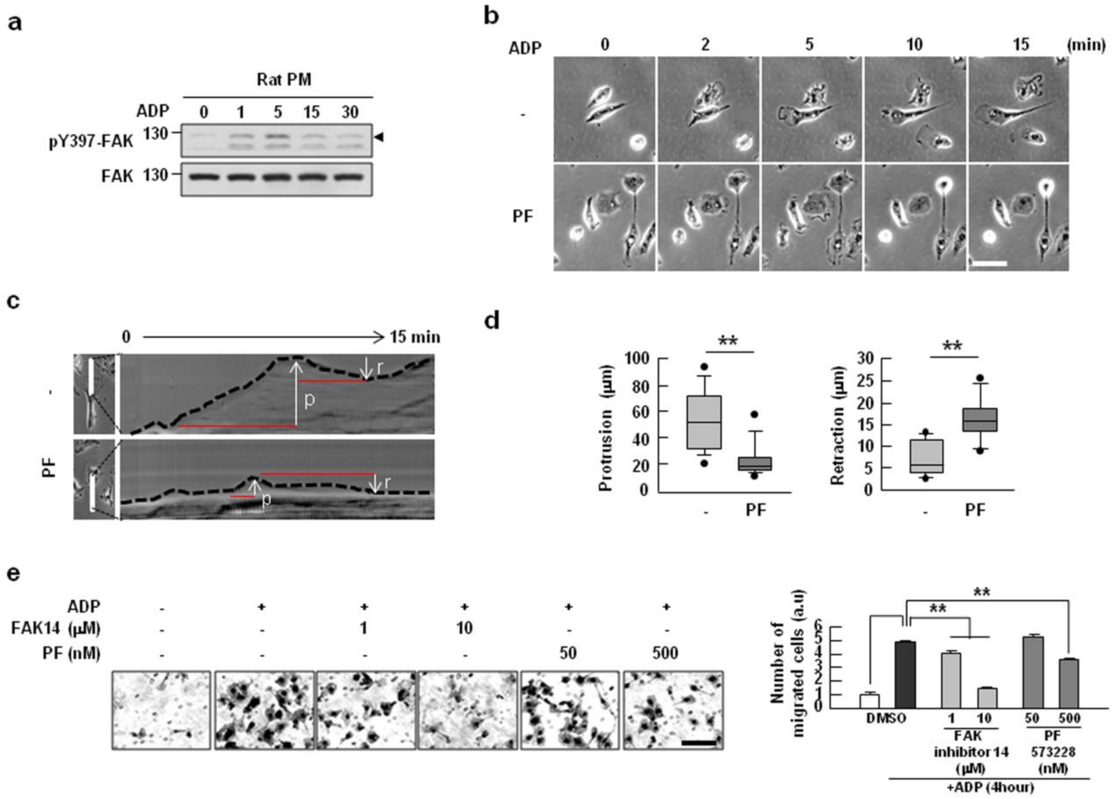

Supplementary Figure 4. FAK inhibitors attenuate ADP-induced microglial movement. (a) Rat microglia were treated with 100 μM ADP for the indicated times. FAK Y397 phosphorylation levels were analyzed by Western blotting using pY397-FAK-specific antibodies. **(b-d)** Rat primary microglia were pretreated with the FAK inhibitor, PF573228 (PF, 500nM) or vehicle (-, DMSO) for 30 min, and then treated with ADP. Images were taken at the indicated times after adding ADP. PF573228-treated microglia failed to stably form lamellipodia and did not move cell body, even in the presence of ADP. Stroboscopic analysis of cell dynamics **(**SACED) showed that PF573228 decreased protrusion and increased retraction (c, d). **(e)** Rat microglia (10^5 cells/well) were seeded onto 8-μm-pore Transwells. Cells were treated with indicated amounts of two different FAK inhibitors, FAK 14 inhibitor or PF573228. Cells were allowed to migrate toward the lower chamber containing 100 μM ADP for 4 hours. Non-migrated cells were removed from the upper surface of Transwells with a cotton swab, and migrated cells on the bottom surface of Transwells were fixed with 4% paraformaldehyde for 20 minutes. Cells were visualized with 1% Crystal violet (Upper panel). The number of cells in nine randomly chosen fields was counted. Data are representative of at least three independent experiments. Values are means ± SEM of three samples. Two-tailed Student t test, $^{**}P<0.01$ in (d). One-way ANOVA with Newman–Keuls post hoc test, $^{**}P<0.01$ in (e). Scale bars, 50 μm in (b), 100 μm in (e).

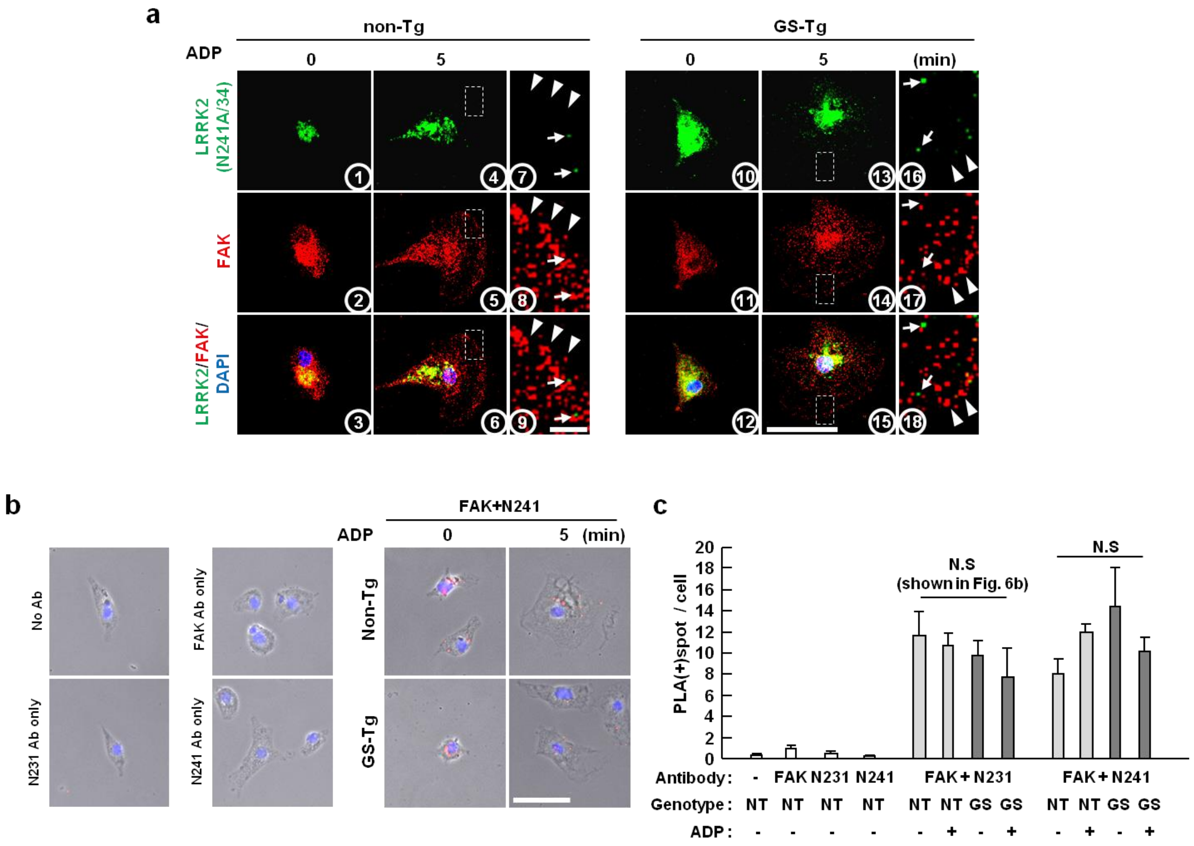

Supplementary Figure 5. LRRK2 interacts with FAK in the cytoplasm. (a, b) Non-Tg and GS-Tg microglia were treated with 100 μM ADP for 5 minutes. **(a)** Cells were stained with specific antibodies against FAK and LRRK2 (N241A/34). Magnified images of boxed areas in '4-6' and '13-15' were shown in '7-9' and '16-18', respectively. Arrows and arrowheads indicate location of LRRK2 and FAK, respectively. **(b, c)** The interaction between FAK and LRRK2 was analyzed in non-Tg and GS-Tg microglia (n=50) using *in situ* PLA, as described in the Methods. We tested two different LRRK2 antibodies, N231B/34 (N231) and N241A/34 (N241). The red PLA spots (representing interactions between the two proteins) were quantified (c). Values are means ± SEM of three separate experiments. N.S, non-significance. Scale bars, 50 μm (a '15', b), 5 μm (a'9').

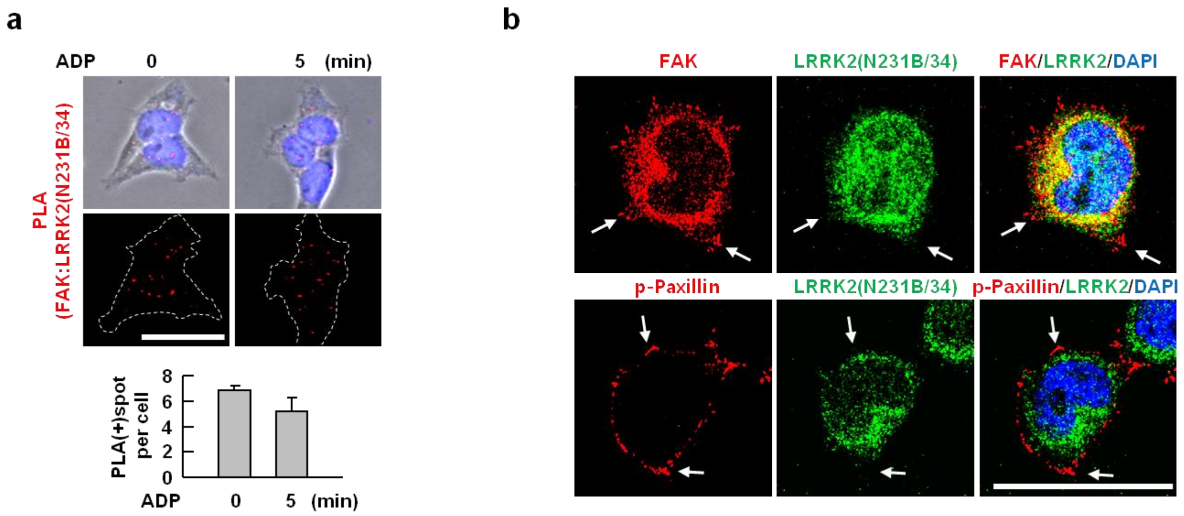

Supplementary Figure 6. Location of FAK and LRRK2 in ADP- or fibronectin-treated HEK cells. (a) Cells were treated with 100 μM ADP for 5 min, and the interaction between FAK and LRRK2 (N231B/34) was examined using *in situ* PLA, as described in the Materials and Methods section (upper panel). The red PLA spots in the upper panel were quantified (lower panel). Dotted lines indicate the cell boundaries. **(b)** Cells were seeded on fibronectin (10 μg/ml)-coated coverslips for 2 h, fixed, and stained for FAK and LRRK2 (upper panel), or for p-paxillin and LRRK2 (lower panel). Arrows indicate focal adhesions where FAK and p-paxillin, but not LRRK2, were located. Scale bars, 50 μm.

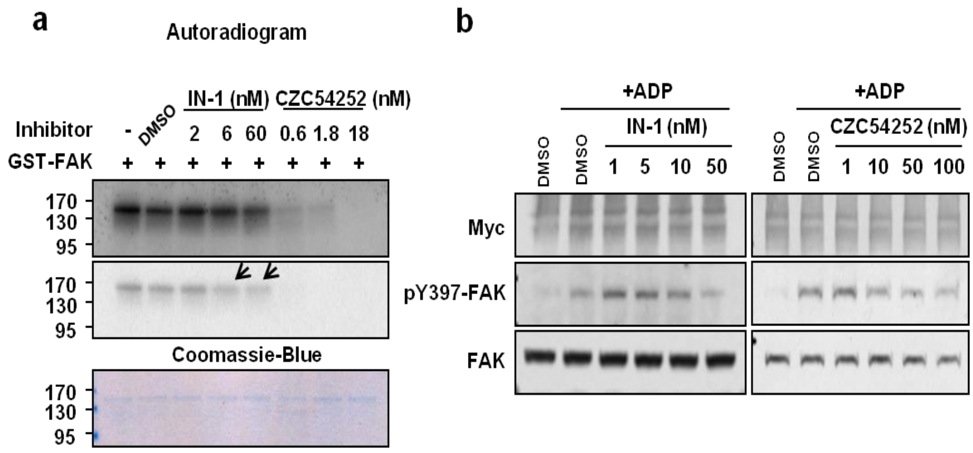

Supplementary Figure 7. Effect of LRRK2 inhibitors, IN-1 and CZC 54254, on FAK activation. **(a)** *In vitro* kinase assays were carried out using recombinant GST-FAK in the absence or presence of the indicated amount of inhibitors, IN-1 and CZC 54252. ^{32}P-labeled FAK was detected in autoradiograms. Upper and middle panels in (a) were obtained with long and short exposure, respectively. Arrows in (a) showed decrease in phosphorylation of FAK in the presence of IN-1. Coomassie blue staining shows the amount of proteins in each reaction mixture (lower panels). **(b)** HEK 293T cells were transfected with Myc-tagged GS-LRRK2, and after forty-eight hours later, cell were pretreated with IN-1 and CZC 54252 for 30 minutes as indicated concentrations followed by ADP (100 μM) treatment for five minutes. The levels of pY397-FAK were analyzed by Western blotting. FAK was used a loading control.

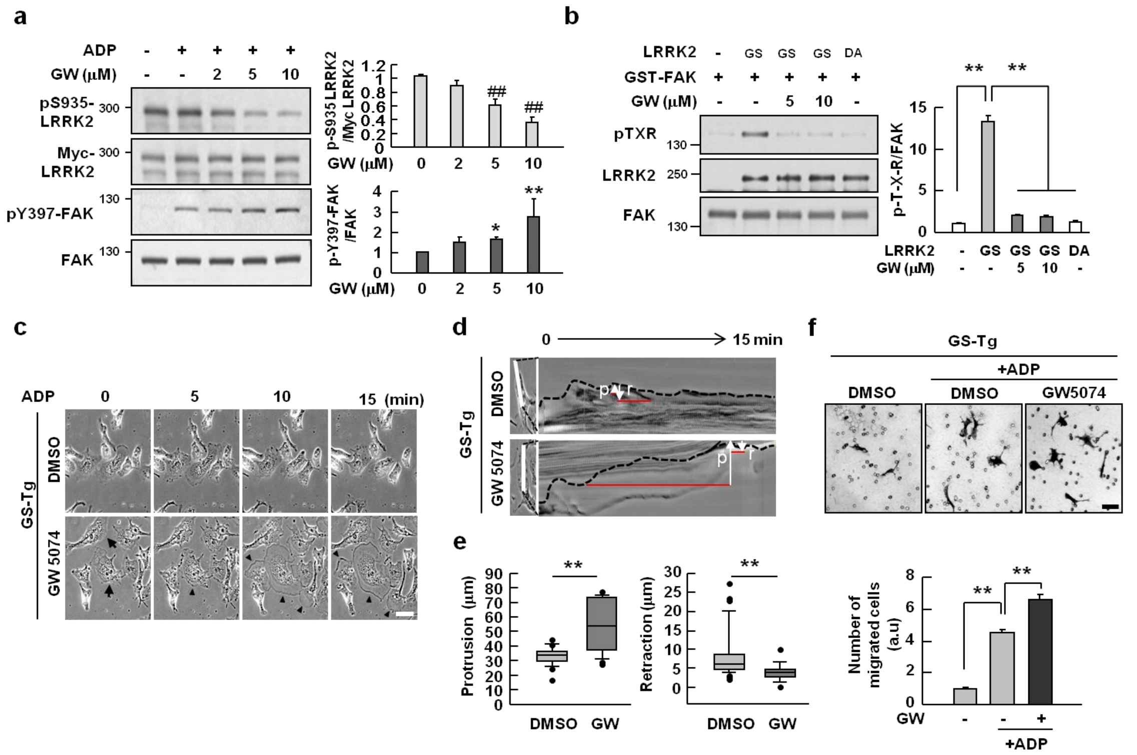

Suppelmentary Figure 8. The LRRK2 kinase inhibitor, GW5074, reduces pTXR-FAK levels and rescues pY397-FAK levels and motility of GS-Tg microglia. (a) HEK 293T cells expressing GS-LRRK2 were treated with ADP (100 μM) for 5 minutes in the presence of the indicated amount of GW5074. Levels of S935-autophosphorylated LRRK2 (pS935-LRRK2) and Y397-autophosphorylated FAK (pY397-FAK) were measured by Western blotting using antibodies specific for pS935-LRRK2 and pY397-FAK, respectively. FAK and Myc were used as loading controls. Band intensities were quantified and plotted. Values are means ± SEM of three separate experiments. **(b)** *In vitro* kinase assays were carried out using recombinant GST-FAK and GS-LRRK2 (GS) or DA-LRRK2 (DA) in the absence or presence of the indicated amount (5 and 10 μM) of GW5074. pTXR-FAK was analyzed and plotted. Values are means ± SEM of three separate experiments. **(c, d, e)** GS-Tg microglia were treated with GW5074 (10 μM) for 30 minutes and then treated with ADP. Microglia adopted a flattened, adhesive morphology in the presence of GW5074 (arrows in c). ADP (100 μM) also vigorously induced formation of stable lamellipodia in the presence of GW5074 (c, arrowheads). Stroboscopic analysis of cell dynamics **(**SACED) showed that GW5074 (n=25) increased protrusion and decreased retraction compared to DMSO (n=25) (d, e). **(f)** Motilities of GS-Tg microglia in the presence of GW5074 (10 μM) were measured using 8-μm-pore Transwells. Values are means ± SEM of three independent experiments. One-way ANOVA with Newman–Keuls post hoc test, *P<0.05, **P<0.01, $^{##}$P<0.01 in (a, b, f). Two-tailed Student t test, **P<0.01 in (e). Scale bar, 50 μm (c)

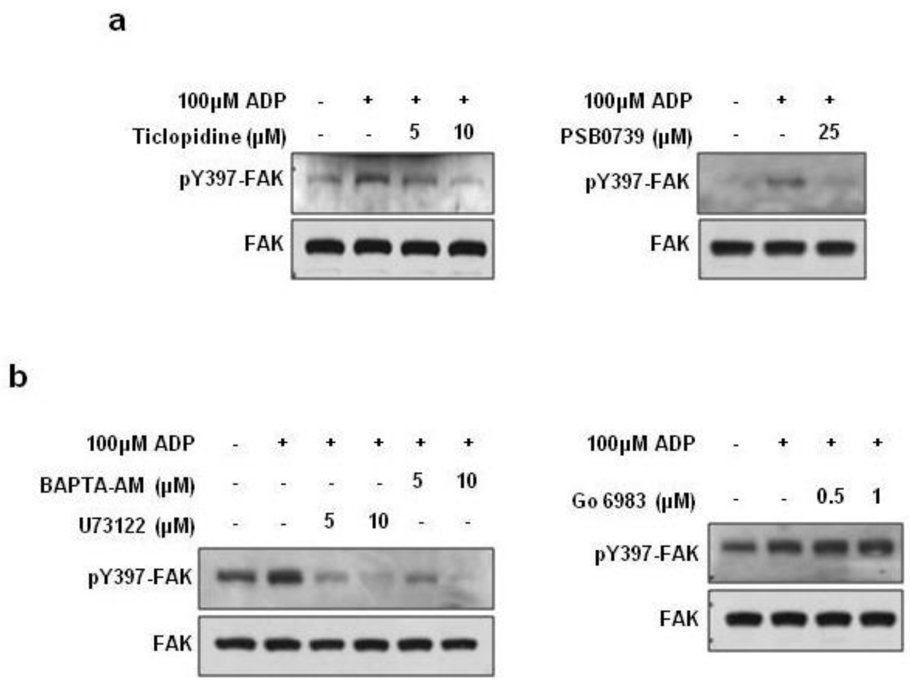

Supplementary Figure 9. Analysis of signaling pathways involved in ADP induced FAK activation. Rat primary microglia were treated with 100 μM ADP in the absence and presence of inhibitors for $P2Y_{12}$ receptor (Ticlopidine and PSB 0739), phospholipase C (U 73122), and PKC (Go 6983), and a Ca^{2+} chelator (BAPTA-AM). FAK Y397 phosphorylation levels were analyzed by Western blotting using pY397-FAK-specific antibodies. FAK was used as a loading control.

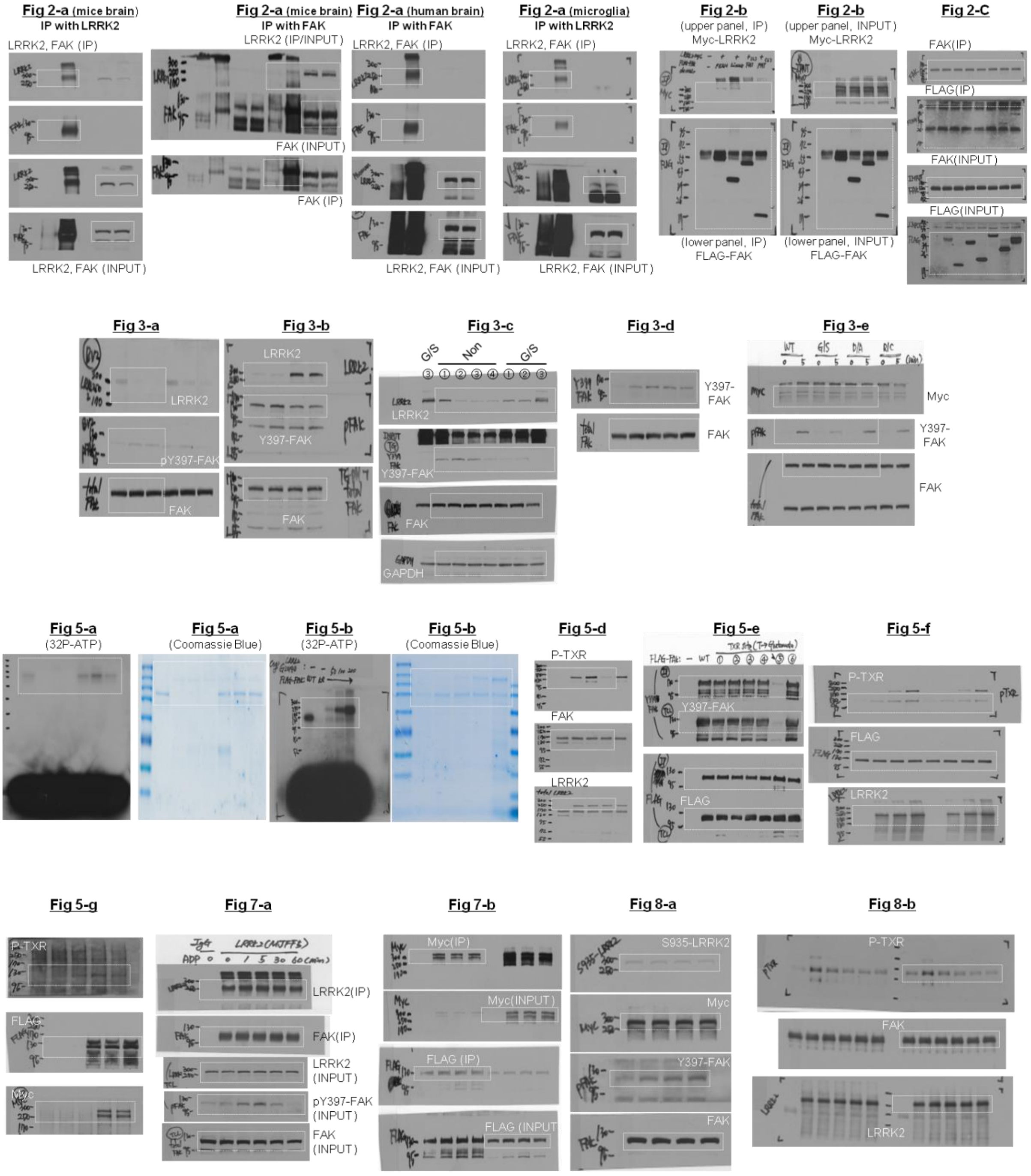

Supplementary Figure 10. Uncropped scans of immunoblots in the main figures

Supplementary Table 1. Primer sequences used for FAK mutagenesis

Mutation	Primer sequences
K454R	5’-AGCTTTGGCGGTTGCAATTAGAACATGTAAAAACTGTACTTCGG-3’
T474A	5’-CTTCAAGAAGCCTTAGCGATGCGTCAGTTTGACCATCCTC-3’
T219E	5’-GGATTCTGTCAAGGCCAAAGAGCTAAGAAAACTGATCC-3’
T227E	5’-CTAAGAAAACTGATCCAACAAGAGTTTAGACAATTTGCCAACC-3’
T284E	5’-AATCAGTTACCTAGAGGACAAGGGCTGCAATCCC-3’
T455E	5’-CGGTTGCAATTAAAGAGTGTAAAAACTGTACTTCGGACAGCG-3’
T474E	5’-CTTCAAGAAGCCTTAGAGATGCGTCAGTTTGACCATCCTC-3’
T979E	5’-TACCAGCCAGCGAGCACCGAGAGATTGAGATGGC-3’
